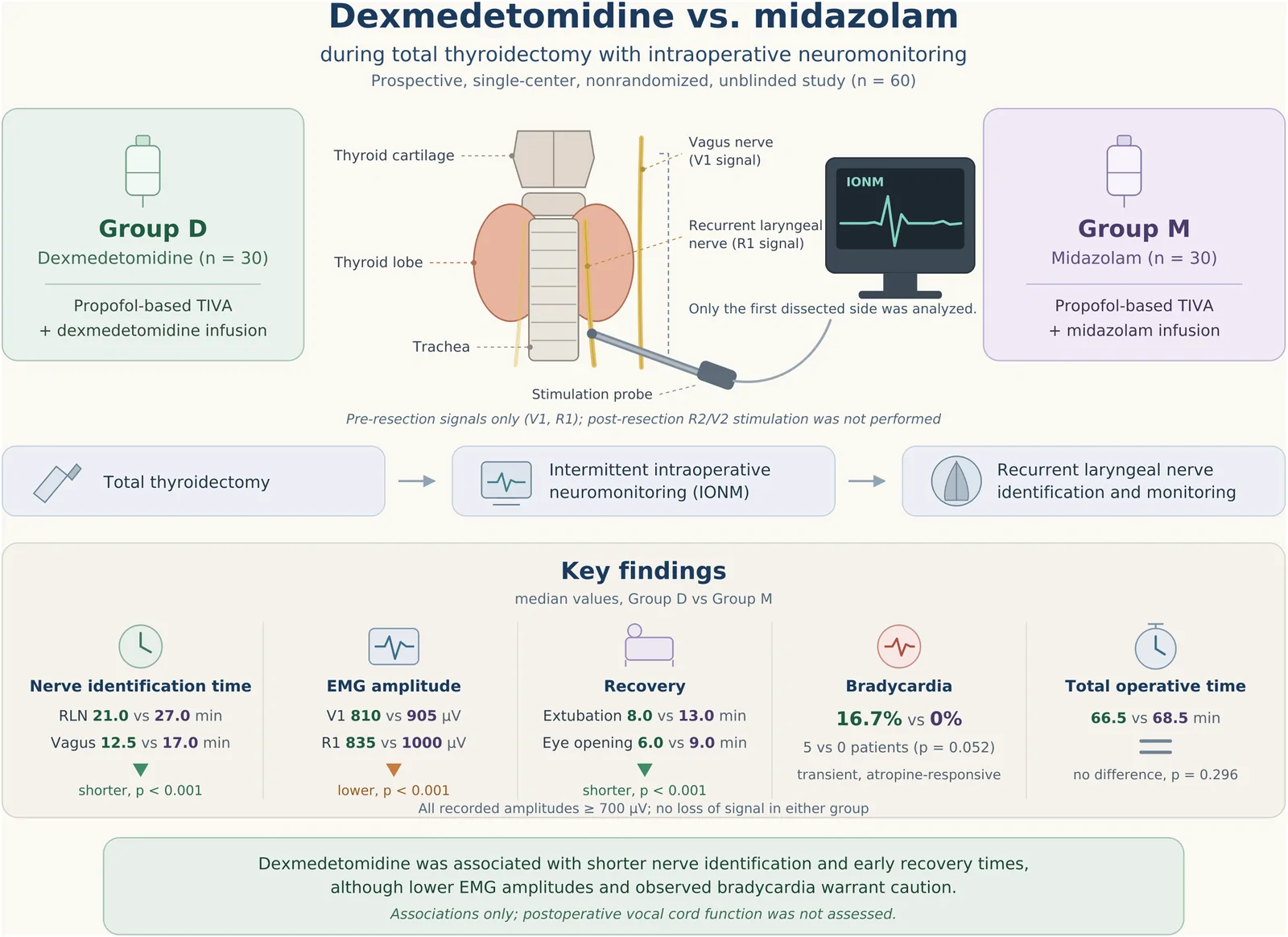# Dexmedetomidine versus midazolam during total thyroidectomy with intraoperative neuromonitoring: A prospective non-randomized study

**DOI:** 10.17305/bb.2026.14894

**Published:** 2026-09-18

**Authors:** Suat Evirgen, Harun Tolga Duran, Bülent Meriç Çam, Mürsel Kahveci, Osman Özgür Kılınç, Şirin Çetin

**Affiliations:** 1 Department of General Surgery, Faculty of Medicine, Amasya University, Amasya, Türkiye; 2 Department of Anesthesiology and Reanimation, Faculty of Medicine, Amasya University, Amasya, Türkiye; 3 Department of Biostatistics, Faculty of Medicine, Amasya University, Amasya, Türkiye

**Keywords:** Thyroid surgery, intraoperative neuromonitoring, total intravenous anesthesia, dexmedetomidine, midazolam

## Abstract

Anesthetic adjuncts may influence intraoperative neuromonitoring (IONM) during thyroidectomy. We compared dexmedetomidine and midazolam as adjuncts to propofol-based total intravenous anesthesia (TIVA). This prospective, single-center, non-randomized, unblinded study included 60 adults undergoing elective total thyroidectomy with intermittent IONM, assigned alternately to dexmedetomidine or midazolam (30 per group). The primary outcome was recurrent laryngeal nerve (RLN) identification time from skin incision on the first dissected side; secondary outcomes were exploratory. Median (interquartile range) RLN identification time was shorter with dexmedetomidine (21.0 [19.0–26.8] versus 27.0 [21.2–31.0] minutes; Hodges–Lehmann median difference, −4.0 minutes; 95% confidence interval [CI], −7.0 to −1.0; *P <* 0.001). Median vagus nerve identification time was also shorter (12.5 versus 17.0 minutes; *P <* 0.001), whereas total operative time did not differ. Pre-resection electromyography (EMG) amplitudes were lower with dexmedetomidine at the vagus nerve (810 versus 905 µV) and RLN (835 versus 1000 µV; both *P <* 0.001), but all recorded amplitudes were ≥700 µV, with no loss of signal. Median extubation and recovery times were shorter (8.0 versus 13.0 and 6.0 versus 9.0 minutes, respectively; both *P <* 0.001). Bradycardia occurred in 16.7% versus 0% of patients (*P* ═ 0.052). Dexmedetomidine was associated with shorter nerve identification and early recovery times, although lower EMG amplitudes and observed bradycardia warrant caution. These findings require randomized confirmation; postoperative vocal cord function was not assessed, precluding conclusions about nerve safety.

## Introduction

One of the most feared complications of thyroid surgery is vocal cord paralysis and persistent hoarseness resulting from recurrent laryngeal nerve (RLN) injury. To mitigate this risk, intraoperative neuromonitoring (IONM) has become an integral component of contemporary thyroid practice [1]. By offering real-time feedback on both the presumed anatomical location and functional integrity of the RLN, IONM supports safer dissection and reduces the likelihood of iatrogenic nerve injury [2, 3]. Prior evidence suggests that, particularly in high-risk settings, IONM use is associated with a lower incidence of permanent RLN injury [4]. Accordingly, many surgeons have incorporated IONM into routine thyroidectomy workflows [5].

Successful and reliable IONM requires anesthetic management compatible with neuromonitoring prerequisites. The key issue is careful handling of neuromuscular blockade. To obtain interpretable electromyography (EMG) signals, the effect of the neuromuscular blocking agent must have sufficiently worn off by the time RLN monitoring begins [6]. For this reason, a single induction dose of a nondepolarizing neuromuscular blocker is commonly used, and repeat dosing is generally avoided during the case [7]. Under conditions of limited neuromuscular blockade, maintaining adequate anesthetic depth becomes essential to prevent movement in response to surgical stimulation [8].

Anesthesia during thyroid surgery can be maintained using either inhalational agents or total intravenous anesthesia (TIVA). However, inhalational anesthetics are used less frequently in IONM-guided cases due to concerns about their interaction with neuromuscular blockade and potential negative impact on EMG signal quality; therefore, TIVA is often preferred [9]. Within TIVA protocols, propofol is widely used due to its rapid onset and predictable recovery profile [10, 11]. Nevertheless, when deeper anesthesia is required, higher propofol doses may increase the risk of adverse effects, most notably hypotension [12]. In this context, adding adjuvant agents is commonly considered to optimize anesthetic depth and hemodynamic stability.

Midazolam and dexmedetomidine are among the most frequently used adjuncts within propofol-based TIVA strategies [13]. Midazolam is a short-acting benzodiazepine with anxiolytic, sedative, and amnestic properties [14]. Despite its rapid onset and short duration, the lack of intrinsic analgesia may limit its ability to blunt surgical nociception, and postoperative delirium or cognitive dysfunction has been reported [15]. Dexmedetomidine is a selective α2-adrenergic receptor agonist that provides sedation with analgesic and sympatholytic effects [16]. However, bradycardia and hypotension remain clinically relevant concerns during its use [17].

Optimal anesthetic management for thyroidectomy performed with IONM remains an area of ongoing discussion. Rather than relying solely on higher-dose propofol to achieve deep anesthesia, incorporating an adjunct may improve hemodynamic control while maintaining adequate anesthetic depth. Dexmedetomidine, through its analgesic-sparing profile, may reduce opioid requirements and contribute to a smoother recovery course. Although midazolam is widely used in everyday practice, data specifically addressing its performance in IONM-guided thyroid surgery are limited.

Therefore, this study aimed to compare dexmedetomidine and midazolam infusions, administered as adjuncts to propofol-based TIVA, with respect to nerve identification times, IONM parameters, intraoperative hemodynamic stability, and early postoperative outcomes in patients undergoing thyroidectomy with IONM. RLN identification time was selected as the primary endpoint because it is a continuous variable measurable in every case, reflecting how readily the nerve is exposed and functionally confirmed under a given anesthetic regimen. Permanent nerve palsy and loss of signal are the most critical outcomes, but they occur in only a small proportion of thyroidectomies, and a study powered for them would require several thousand patients. Identification time was therefore used as a feasible surrogate for intraoperative conditions rather than as a direct measure of nerve safety.

## Materials and methods

This was a prospective, non-randomized comparative study conducted in the departments of general surgery and anesthesiology of Amasya University Faculty of Medicine, Amasya, Türkiye. Patients were recruited consecutively between March 2025 and July 2025, with data collection completed in July 2025. Because treatment allocation was not under experimental control, the study is reported in accordance with the Strengthening the Reporting of Observational Studies in Epidemiology (STROBE) statement for observational studies. This study was registered at ClinicalTrials.gov (NCT07195214).

### Group allocation and blinding

Allocation to the two groups was not randomized. Patients were assigned in alternating order according to their position on the operating list, with odd-numbered patients receiving dexmedetomidine and even-numbered patients midazolam. Because the sequence was determined solely by the operating list, no patient, disease, or operative characteristic influenced the assignment. However, the allocation sequence was foreseeable and not concealed from the clinical team. No placebo infusion was used, and neither the anesthesiology team nor the operating surgeon was blinded to the administered adjunct. Intraoperative time points were recorded by the neuromonitoring technician, who did not participate in the anesthetic or surgical procedures. No other outcome assessment was blinded. Although the technician responsible for logging these intraoperative time points was unaware of which adjunct infusion each patient was receiving, the event that triggered each recorded time — the surgeon’s determination that the vagus nerve or RLN had been visually identified and functionally confirmed by stimulation — was made by the unblinded operating surgeon, who then signaled the technician to note the time.

### Study population

Patients aged 18–65 years with American Society of Anesthesiologists (ASA) physical status I–II scheduled for elective total thyroidectomy for benign nodular goiter, multinodular goiter (MNG), or thyroid malignancy were eligible. Exclusion criteria included preoperative vocal cord paralysis; severe cardiovascular, renal, or hepatic dysfunction; known allergy to the study medications; and use of sedative or analgesic drugs within 24 h before surgery. No patient in either group had retrosternal extension of the gland or required additional neck dissection beyond total thyroidectomy. All operations were primary procedures; no patient had undergone previous thyroid or neck surgery, and no patient had Graves’ disease. Nine patients (four in Group D, five in Group M) had papillary thyroid carcinoma; no central or lateral neck dissection was performed in any case.

### Anesthesia and monitoring

Standard monitoring (electrocardiography, noninvasive blood pressure, and pulse oximetry) was applied to all patients. After preoxygenation with 100% oxygen, anesthesia was induced with intravenous propofol (2 mg/kg) and fentanyl (2 µg/kg). To facilitate tracheal intubation, a single dose of rocuronium bromide (0.4 mg/kg) was administered (limited to a total dose of 30 mg to minimize interference with IONM). Intubation was performed using a nerve monitoring endotracheal tube with integrated EMG electrodes positioned at the level of the vocal cords (Technocath Technotech, Yalova, Türkiye). Tube position was verified by direct laryngoscopy (visualizing vocal cord abduction) and by confirming acceptable electrode impedance values on the neuromonitoring system.

Anesthesia was maintained in both groups using a propofol-based TIVA protocol, with no inhalational agents used. Mechanical ventilation was set to deliver a tidal volume of 6–8 mL/kg of ideal body weight, with respiratory rate adjusted to maintain end-tidal CO_2_ between 35 and 40 mmHg. Depth of anesthesia was guided by bispectral index (BIS) monitoring and maintained between 40 and 60 throughout the procedure.

### Study groups

Group D (Dexmedetomidine): Before induction, dexmedetomidine was administered as a loading infusion over 10 min (0.5 µg/kg as per protocol), followed by a maintenance infusion titrated to 0.4–0.7 µg/kg/h until the end of surgery. Anesthesia was maintained with propofol infusion (4–8 mg/kg/h), which was modified according to the BIS value. Analgesia was provided with remifentanil infusion (0.1–0.2 µg/kg/min). Supplemental propofol boluses (20–30 mg) were administered when clinically required.

**Group M (Midazolam):** Patients received an intravenous loading dose of midazolam (0.03 mg/kg) before induction, followed by a continuous infusion of 0.03–0.1 mg/kg/h post-induction. Anesthesia was maintained with a propofol infusion (4–8 mg/kg/h), adjusted based on BIS values. Analgesia was sustained with a remifentanil infusion (0.1–0.2 µg/kg/min), consistent with Group D.

Both groups received a single intubating dose of rocuronium, with no additional neuromuscular blocking agents administered during the operation. While the dosage and agent were identical, the interval between intubation and the first stimulation was not individually recorded. Since nerve identification occurred earlier in Group D, this interval might have been shorter for that group. Neuromuscular recovery was not objectively confirmed with train-of-four monitoring before stimulation. Therefore, a higher degree of residual neuromuscular blockade in Group D cannot be ruled out as a potential factor contributing to the lower observed EMG amplitudes (see Study Limitations). Hemodynamic events and treatments were predefined: hypotension (systolic blood pressure below 90 mmHg) was managed with intravenous ephedrine (5–10 mg), and bradycardia (heart rate below 50 beats/min) with intravenous atropine (0.5 mg). All episodes of hypotension/bradycardia and pharmacological interventions were meticulously recorded.

### Surgical procedure and intermittent IONM protocol

All operations were performed by the same surgeon, experienced in IONM, using a standardized intermittent IONM approach. After thyroid lobe mobilization, the vagus nerve within the carotid sheath was identified and stimulated to obtain baseline vocal cord EMG responses. The RLN was then dissected from the inferior pole, visually identified, and stimulated with a probe to confirm functional integrity. According to the International Neural Monitoring Study Group’s terminology, the reported responses are the initial vagal (V1) and recurrent laryngeal (R1) signals obtained prior to lobe resection. Post-resection stimulation for R2 and V2 responses was not performed or obtained in this study. Vocal muscle action potentials were recorded using the NIM-Response 3.0 neuromonitoring system (Medtronic Xomed, Jacksonville, FL, USA). A monopolar probe was consistently used, with a pulse duration of 100 µs and a stimulation frequency of 4 Hz. Mapping and initial localization were conducted at up to 2.0 mA. Once the nerve was visually identified, the evoked response entered into the analysis was recorded at a fixed current of 2.0 mA for every patient, ensuring direct comparability of amplitudes between groups and between the two nerves. As total thyroidectomy is a bilateral procedure, each patient underwent sequential vagus and RLN monitoring on both sides following the same protocol. To avoid pooling non-independent, within-patient observations and to maintain unambiguous timing and amplitude outcomes, only the first side dissected in each operation was used for the analyses presented here. The choice of the first side operated on followed the surgeon’s usual practice and was not determined by the study protocol. The contralateral side was monitored identically but was not included in the primary dataset.

### Outcomes and definitions

The primary outcome was RLN identification time. All other measures listed below were secondary. Both identification times were measured from the skin incision, chosen as an unambiguous, objectively documented event, rather than from the onset of nerve dissection, which lacks a single, well-defined starting point.

- **Vagus nerve identification time (min):** Time from skin incision to surgical identification of the vagus nerve on the first dissected side, and acquisition of the first EMG response after stimulation.
- **RLN identification time (min):** Time from skin incision to visual identification of the RLN on the first dissected side, and functional confirmation by stimulation after thyroid lobe mobilization and dissection of the tracheoesophageal groove.
- **EMG signal amplitude (µV):** On the first dissected side, the peak-to-peak voltage of the evoked vocal muscle response to the first vagal (V1) and recurrent laryngeal (R1) stimulation, recorded at a fixed stimulation current. For context, two further definitions from standard IONM terminology are provided. Loss of signal was defined as an amplitude below 100 µV on stimulation at 1–2 mA, excluding equipment-related and technical causes; this was assessed in our data (see Results). An amplitude reduction of at least 50% accompanied by a latency increase of at least 10%, comparing pre- and post-resection responses, is conventionally termed an adverse EMG change or warning event. Since only the pre-resection response (V1/R1) was analyzed in this study, this second definition is provided for terminological context only and was not evaluated as an outcome here.
- **Intraoperative hemodynamics***:* Mean arterial pressure and heart rate were recorded at five predefined time points: induction, intubation, vagus nerve stimulation, RLN stimulation, and extubation. For each patient, values recorded at these time points were averaged to yield a single patient-level mean heart rate and mean arterial pressure. The “mean heart rate” and “mean arterial pressure” reported in the Results represent these patient-level means, summarized across patients as group median (interquartile range). Episodes of bradycardia (heart rate below 50 beats/min) and hypotension (systolic blood pressure below 90 mmHg) were documented along with the use of atropine and ephedrine.
- **Extubation time (min):** Time from cessation of anesthesia to removal of the endotracheal tube.
- **Recovery time (min):** Time from cessation of anesthesia to the first eye opening in response to a verbal command.
- **Early postoperative outcomes:** These included postoperative nausea and vomiting (PONV) within the first 24 h; pain, assessed with a visual analogue scale (VAS, 0–10) at the second postoperative hour; the need for rescue opioid analgesia within the first two postoperative hours; and agitation or delirium. These outcomes were assessed by the same anesthesiology and surgical team, who were not blinded to group allocation, using standard clinical observation and patient report rather than a validated sedation-agitation or delirium scale. Antiemetic and rescue analgesic treatment were administered at the treating clinician’s discretion, not according to a standardized protocol.

Postoperative vocal cord function was not assessed. Consequently, the study focused solely on neuromonitoring performance and early recovery, providing no information regarding nerve outcome.

### Statistical analysis

Statistical analyses were conducted using IBM SPSS Statistics for Windows, Version 25.0 (IBM Corp., Armonk, NY, USA). Normality of continuous variables was assessed using the Shapiro–Wilk test. As every continuous variable deviated from normality in at least one group, all continuous data are presented as median (interquartile range) and were compared using the Mann–Whitney *U* test. Categorical variables are presented as number (percentage) and compared using the chi-square test or Fisher’s exact test, as appropriate. A two-sided P value <0.05 was considered statistically significant. The confirmatory analysis was restricted to RLN identification time; all other comparisons were secondary and exploratory, with no adjustment for multiple testing, so the corresponding P values should be interpreted as descriptive. For the primary outcome, the between-group difference is additionally reported as the Hodges–Lehmann estimator of the median difference with its 95% confidence interval, calculated using the normal approximation to the Mann–Whitney U distribution.

### Ethical statement

The study protocol received approval from the Clinical Research Ethics Committee of Amasya University (meeting no. 03, decision no. 2025/09, dated February 20, 2025) and adhered to the principles of the Declaration of Helsinki. All participants received comprehensive study information and provided written informed consent prior to enrollment.

### Sample size and power analysis

The sample size was determined using RLN identification time as the primary outcome. Lacking directly comparable data, we based our calculation on a five-minute between-group difference in RLN identification time, assuming a standard deviation of 6.4 min. This corresponds to a standardized difference (Cohen’s d) of 0.78, which we considered the smallest clinically relevant effect. Both this threshold and the assumed standard deviation were based on the operating surgeon’s clinical judgment and departmental experience with nerve identification in similar cases, as no validated data specific to this comparison were available.

We acknowledge that the sample size calculation, based on a two-sample *t*-test, does not precisely align with the nonparametric test ultimately used for analysis. The buffer between the calculated minimum sample size and the number actually enrolled was intended to accommodate the slight loss of efficiency associated with nonparametric methods.

An *a priori* power analysis, assuming a two-tailed independent-samples *t*-test with α set at 0.05 and power (1-β) at 0.80, indicated a minimum required sample size of 27 patients per group. To account for potential data loss and perioperative exclusions, the target sample size was increased to 30 patients per group, totaling 60 patients. The observed difference for the primary outcome exceeded our initial assumption, confirming the study was adequately powered for its research question.

## Results

A total of 60 patients were included (Group D: *n* ═ 30; Group M: *n* ═ 30). Age, sex distribution, body mass index, and total operative time were comparable between groups (all *P* > 0.05). No patients were withdrawn after enrollment (Table 1).

**Table 1:** Demographic and perioperative characteristics of the study groups

**Characteristic**	**Group D (*n* ═ 30)**	**Group M (*n* ═ 30)**	***P* value**
Age (years)	46.5 (33.2–56.2)	47.5 (35.8–57.5)	0.554
Sex (female/male), *n*	21/9	22/8	0.774
BMI (kg/m^2^)	28.3 (24.5–31.5)	27.7 (25.5–29.9)	0.723
Total operative time (min)	66.5 (59.0–73.8)	68.5 (62.0–79.2)	0.296

Continuous variables are presented as median (interquartile range) and were compared using the Mann-Whitney *U* test. Sex was compared using the chi-square test. **Abbreviations**: BMI, body mass index; Group D, dexmedetomidine group; Group M, midazolam group.

### IONM outcomes

IONM outcomes are summarized in Table 2. Vagus nerve identification was significantly faster in Group D, with a median of 12.5 min (interquartile range [IQR] 11.0–15.8) compared to 17.0 min (15.0–19.8) in Group M (*P <* 0.001). Similarly, RLN identification was faster in Group D, at 21.0 min (19.0–26.8) vs 27.0 min (21.2–31.0) (Hodges–Lehmann median difference -4.0 min, 95% confidence interval [CI] −7.0 to −1.0; *P <* 0.001). Total operative time was similar between groups (*P ═* 0.296), indicating the difference was confined to nerve exposure and functional confirmation, not the entire operation.

**Table 2:** Intraoperative neuromonitoring parameters of the study groups

**Parameter**	**Group D (*n* ═ 30)**	**Group M (*n* ═ 30)**	***P* value**
Vagus nerve identification time (min)	12.5 (11.0–15.8)	17.0 (15.0–19.8)	<0.001
RLN identification time (min)	21.0 (19.0–26.8)	27.0 (21.2–31.0)	<0.001
Vagus signal amplitude, V1 (µV)	810 (780–868)	905 (852–958)	<0.001
**Vagus signal amplitude range, V1 (µV)**	**710–930**	**800–970**	**—**
RLN signal amplitude, R1 (µV)	835 (745–930)	1000 (888–1070)	<0.001
**RLN signal amplitude range, R1 (µV)**	**700–1070**	**780–1140**	**—**
**Loss of signal, vagus, *n* (%)**	**0 (0)**	**0 (0)**	**NA**
**Loss of signal, RLN, *n* (%)**	**0 (0)**	**0 (0)**	**NA**

Data are presented as median (interquartile range). All comparisons were performed using the Mann-Whitney *U* test. Loss of signal was defined as an amplitude below 100 µV. NA indicates that no events occurred in either group, precluding a meaningful statistical comparison. **Abbreviations**: Group D, dexmedetomidine group; Group M, midazolam group; NA, not applicable; R1, initial recurrent laryngeal nerve signal before resection; RLN, recurrent laryngeal nerve; V1, initial vagus nerve signal before resection.

Conversely, EMG amplitudes were lower in Group D. Vagal responses were 810 µV (IQR 780–868) in Group D compared to 905 µV (852–958) in Group M (*P <* 0.001). RLN responses were also lower in Group D, at 835 µV (745–930) vs 1000 µV (888–1070) (*P <* 0.001). While consistent, this difference was not substantial. The lowest amplitude recorded across the cohort was 700 µV, seven times the 100 µV threshold for loss of signal, and diagnostic-quality traces were obtained for every patient in both groups. No loss of signal occurred. Adverse EMG changes were not evaluated, as only pre-resection responses were analyzed.

### Hemodynamic and recovery outcomes

Hemodynamic parameters and intraoperative events are presented in Table 3. Mean arterial pressure was similar between groups, at 77.0 mmHg (IQR 68.2–92.5) in Group D and 81.5 mmHg (75.5–93.5) in Group M (*P ═* 0.657). The incidence of hypotension requiring ephedrine did not differ (4 vs. 3 patients; *P ═* 1.000, Fisher’s exact test). Heart rate was lower in Group D, with a median of 58.5 beats/min (56.0–80.8) compared to 72.5 beats/min (60.0–90.0) (*P ═* 0.002). Bradycardia requiring atropine occurred in five patients (16.7%) in Group D and none in Group M; this comparison did not reach conventional significance (*P ═* 0.052, Fisher’s exact test).

**Table 3:** Hemodynamic and postoperative outcomes of the study groups

**Parameter**	**Group D (*n* ═ 30)**	**Group M (*n* ═ 30)**	***P* value**
Mean heart rate (beats/min)	58.5 (56.0–80.8)	72.5 (60.0–90.0)	0.002
Bradycardia (HR <50 bpm), *n* (%)	5 (16.7)	0 (0)	0.052
Mean arterial pressure (mmHg)	77.0 (68.2–92.5)	81.5 (75.5–93.5)	0.657
Hypotension (SBP <90 mmHg), *n* (%)	4 (13.3)	3 (10.0)	1.000
Extubation time (min)	8.0 (7.0–9.8)	13.0 (11.0–14.0)	<0.001
Recovery time (min)	6.0 (5.0–7.8)	9.0 (8.0–10.0)	<0.001
PONV (24 h), *n* (%)	3 (10.0)	9 (30.0)	0.053
VAS pain score at 2 h (0–10)	3.0 (2.0–4.8)	4.0 (3.0–5.0)	0.440
Rescue opioid within 2 h, *n* (%)	5 (16.7)	10 (33.3)	0.136
Agitation or delirium, *n* (%)	0 (0)	4 (13.3)	0.112

Continuous variables are presented as median (interquartile range) and were compared using the Mann–Whitney *U * test. Mean heart rate and mean arterial pressure are patient-level means across five predefined intraoperative time points (induction, intubation, vagus stimulation, RLN stimulation, and extubation), summarized here as the median (interquartile range) across patients. Categorical variables are presented as n (%) and were compared using the chi-square test, or Fisher’s exact test when any expected cell count was below five. **Abbreviations**: Bpm, beats per minute; Group D, dexmedetomidine group; Group M, midazolam group; HR, heart rate; PONV, postoperative nausea and vomiting; RLN, recurrent laryngeal nerve; SBP, systolic blood pressure; VAS, visual analogue scale.

Recovery was faster in Group D. Extubation took a median of 8.0 min (IQR 7.0–9.8) vs 13.0 min (11.0–14.0) in Group M. The interval to first eye opening on command was 6.0 min (5.0–7.8) vs 9.0 min (8.0–10.0). Both differences were highly significant (*P <* 0.001 for each).

Within the first 24 postoperative hours, PONV occurred in three patients (10%) in Group D and nine patients (30%) in Group M, a difference approaching statistical significance (*P ═* 0.053). Pain at the second postoperative hour was similar, with a median VAS of 3.0 (IQR 2.0–4.8) in Group D and 4.0 (3.0–5.0) in Group M (*P ═* 0.440). Rescue opioid was required by five patients (16.7%) in Group D vs ten (33.3%) in Group M (*P ═* 0.136). No agitation or delirium was observed in Group D, whereas four patients (13.3%) in Group M exhibited these symptoms (*P ═* 0.112, Fisher’s exact test).

## Discussion

This study found that adding dexmedetomidine to propofol-based TIVA was associated with shorter nerve identification times during thyroidectomy with IONM. This aligns with previous reports suggesting dexmedetomidine can improve operating conditions in upper airway procedures [18]. A pharmacological explanation is plausible: dexmedetomidine, a selective α2-adrenergic receptor agonist with central actions, reduces sympathetic outflow via locus coeruleus activity, thereby decreasing norepinephrine release and attenuating stress-related cardiovascular responses. It also provides analgesia by modulating spinal neurotransmission involving substance P and glutamate [18]. It is reasonable to hypothesize that fewer hemodynamic surges and a calmer operative field facilitate meticulous nerve dissection. However, we did not record surgical field quality, surgeon satisfaction, patient movement, or other objective indices of operating conditions. Therefore, the link between these pharmacological properties and the shorter identification times is a hypothesis generated by this study, not a supported conclusion, and warrants direct testing.

Several alternative, non-pharmacological explanations for the shorter identification times cannot be excluded. As the surgeon was aware of group allocation and personally determined the moment of identification, performance bias is possible, as detailed in “Study Limitations.” The groups may also have differed in intraoperative opioid or propofol requirements, gland pathology, or anatomical complexity, none of which were fully characterized or balanced by design. Because allocation alternated by operating list position, list order and any associated learning or fatigue effects over the course of an operating day cannot be ruled out as confounders. Residual neuromuscular blockade, discussed previously in relation to amplitude findings, could also affect the ease of eliciting a confirmatory EMG response and thus the recorded identification time. Any claim that dexmedetomidine produced better surgical or operating conditions would require independent, objective measurement of those conditions, which this study did not perform. The greater need for rescue opioid in the early postoperative period in the midazolam group, while consistent with benzodiazepines’ lack of intrinsic analgesic activity and limited sympatholytic effect [19], did not reach statistical significance and requires similar caution.

The effect size warrants separate comment. The median difference was 4.5 min for the vagus nerve and 6 min for the RLN, representing approximately a 25% reduction in the time needed to expose and functionally confirm each nerve. Total operative time remained unchanged, indicating the time saving was absorbed within the operation. The clinical significance of this depends on the context. While unlikely to alter the course of a straightforward thyroidectomy, earlier nerve identification may be beneficial in high-volume operating lists or in reoperative or large substernal glands where dissection is prolonged and the surgeon operates longer at the highest risk point. Importantly, this finding is not evidence of a safety benefit. We did not examine vocal cords post-surgery, recorded no loss of signal in either group, and a cohort of 60 patients lacks the power to detect differences in events occurring in a small percentage of thyroidectomies. Thus, shorter identification times should not be interpreted as a proxy for reduced nerve injury risk; they describe the surgical process, not nerve outcomes.

The amplitude data require a more careful interpretation than the identification times. Dexmedetomidine has been reported to suppress motor and somatosensory evoked potential amplitudes [20, 21], likely by enhancing inhibitory pathways within the central nervous system [22]. Our results are consistent with this. Amplitudes were lower in the dexmedetomidine group at both stimulation sites, with highly significant differences. Since every response was recorded with the same stimulation current, probe, and pulse settings, the comparison is unlikely to reflect differences in stimulation technique. The neuromuscular blocking regimen was also identical in dose and timing. However, because nerve identification occurred earlier in Group D, stimulation likely occurred closer to rocuronium administration. Therefore, residual neuromuscular blockade, rather than a direct effect of dexmedetomidine on the evoked response, cannot be excluded as a contributor to the lower observed amplitudes (see “Study Limitations”). While a single significant comparison could be dismissed as chance, the consistent effect on both the vagus nerve and RLN, in the direction predicted by pharmacology, cannot be, and argues against presenting dexmedetomidine as uniformly superior for neuromonitoring.

Nonetheless, its practical importance appears limited in our studied population: the lowest recorded amplitude was 700 µV (seven times the loss-of-signal threshold), no value fell below 300 µV, diagnostic-quality traces were obtained in every patient, and monitoring-guided decisions were never compromised. The critical question is what happens at the margins, such as in a thin or previously operated nerve, or when baseline responses are already near the alert threshold. Our cohort cannot address this situation as it did not arise, and we flag this as a caution rather than dismiss it. The broader message remains that carefully titrated intravenous regimens preserve signal quality during IONM [23]. Within this context, midazolam produced larger amplitudes and dexmedetomidine shorter identification times. Neither advantage is substantial enough to singularly dictate the choice of adjunct.

Bradycardia is a counterweight to these advantages, occurring in one in six patients receiving dexmedetomidine but in none of those receiving midazolam. While this comparison falls short of conventional significance—unsurprising given the absence of events in one group—the direction aligns with the drug’s sympatholytic profile and earlier findings [24]. A five-to-zero split should not be dismissed by clinicians based solely on a P value. In our patients, episodes were transient, responsive to standard measures (atropine), and required no treatment escalation beyond routine care. Neither mean arterial pressure nor the incidence of hypotension differed between groups.

This reassurance, however, must be considered within the study population: ASA I–II patients aged 18–65 years without severe cardiovascular disease. Patients with conduction abnormalities, symptomatic bradyarrhythmia, or ongoing beta-blocker or calcium-channel-blocker therapy are precisely those in whom an α2-agonist infusion is least forgiving. An incidence of 16.7% in a low-risk cohort is not trivial and should temper any recommendation of dexmedetomidine as a default adjunct. The lower observed rate of PONV with dexmedetomidine did not reach statistical significance, but the direction is consistent with its opioid-sparing properties and previously reported favorable emetic profile [25].

Another clinically relevant finding was the association between dexmedetomidine use and shorter early recovery times. Dexmedetomidine’s pharmacological profile allows for relatively prompt emergence after discontinuation, whereas midazolam’s tissue redistribution and active metabolites can prolong recovery [26, 27]. Evidence from critically ill populations, including the Safety and Efficacy of Dexmedetomidine Compared With Midazolam (SEDCOM) trial, shows that dexmedetomidine can shorten ventilation duration and improve recovery quality compared with midazolam [28]. Consistent with this evidence, patients receiving dexmedetomidine were extubated sooner and opened their eyes to command earlier, with median differences of five and three minutes, respectively. Additionally, dexmedetomidine has been associated with a reduced risk of postoperative delirium [29]. The agitation and delirium symptoms observed only in the midazolam group support these prior observations, though four events in sixty patients do not constitute a statistically significant difference, and the study was not designed to establish causality. The lower incidence of PONV in the dexmedetomidine group may be multifactorial, potentially related to reduced opioid requirements and other proposed antiemetic mechanisms [30]. All these were secondary endpoints, assessed without blinding or adjustment for multiple comparisons; they are best interpreted as a coherent, biologically plausible pattern warranting confirmation rather than as established effects.

### Study limitations

Several limitations qualify these findings. First, the absence of true randomization. Alternating assignment by operating list position mitigated obvious selection bias, as no clinical characteristics influenced allocation. However, the sequence was predictable and unconcealed, so an influence on the order in which patients were listed cannot be ruled out. While measured baseline variables were comparable, this does not preclude unmeasured confounding. Our findings therefore describe associations, and no causal claims should be drawn.

Second, the study was unblinded. Neither the anesthesiology team nor the surgeon could be unaware of the adjunct used. The primary endpoint—a time interval determined by the operator—is a judgment made at the operating table, not a machine reading. Surgeon expectation could therefore have shortened recorded times in one group. Postoperative endpoints relying on observation rather than measurement—PONV, pain scoring, rescue opioid decisions, and agitation—are susceptible to the same bias, as they were assessed by the unblinded team. Antiemetic and analgesic management were also left to clinician discretion rather than standardized protocols, introducing another potential source of between-group variation independent of the study drug. A partial safeguard was in place: the technician logging intraoperative time points was unaware of the adjunct each patient received, preventing selective rounding or post hoc adjustment of documented times. However, this does not resolve the more fundamental expectation bias, as the judgment triggering each recorded time was made by the unblinded surgeon, not an independent, blinded observer. For this reason, we describe observed differences in identification time and all other findings as associations rather than evidence of a causal effect. A design with masked infusions and independent, blinded timing of intraoperative milestones would address this concern.

A third limitation is multiplicity. Beyond the primary endpoint, we compared numerous secondary variables without correction for multiple testing. At a nominal alpha of 0.05, at least one spurious significant result would be expected by chance. Several secondary P values are close to the conventional threshold and would not survive formal adjustment; accordingly, these secondary analyses are presented as exploratory. A further limitation concerns the validity of the primary endpoint itself. Both identification times were measured from skin incision rather than the start of nerve dissection. While skin incision is unambiguous and objectively documented, the onset of dissection lacks a single well-defined moment. Consequently, the measured interval includes exposure and thyroid mobilization, as well as the immediate dissection preceding nerve identification. This means it reflects operative difficulty and surgical technique in addition to any effect of the anesthetic adjunct; a difference in this composite interval cannot be attributed solely to the study drug. To our knowledge, nerve identification time has not been formally validated as a measure of IONM quality in prior literature. As noted in the Introduction, we used it as a feasible and measurable surrogate rather than a validated outcome, which should be borne in mind when interpreting its clinical significance.

The most significant oversight is the absence of post-surgical vocal cord function examination. This study, therefore, only describes neuromonitoring behavior under two anesthetic adjuncts and offers no insight into nerve outcomes. It should not be interpreted as such, and future confirmatory studies must include laryngoscopic assessments before discharge and at follow-up. Consequently, the lack of signal loss in either group holds little weight, as 60 patients are insufficient to exclude differences in events that occur in a small percentage of thyroidectomies.

Several further limitations warrant mention. The study’s single-center design, conducted by one experienced surgeon, enhances internal consistency but compromises external validity. Thus, the findings may not apply to surgeons with varying experience or units employing different IONM protocols. The sample size, calculated for nerve identification time, is inadequate for clinically relevant endpoints. The cohort comprised benign nodular goiter, MNG, and malignancy; however, the varying dissection difficulty and nerve exposure associated with these conditions were not recorded in a manner allowing stratified analysis. No patient presented with retrosternal extension, required dissection beyond total thyroidectomy, had prior thyroid or neck surgery, or suffered from Graves’ disease, thereby eliminating four potential sources of variable dissection difficulty. While malignancy (papillary thyroid carcinoma) was identified and balanced between groups (four cases in Group D, five in Group M), the distribution of benign nodular goiter vs multinodular goiter, gland size, and thyroiditis among the remaining patients were not systematically recorded. Therefore, we cannot rule out that unmeasured differences in benign gland pathology contributed to the observed between-group difference in nerve identification time. As these variables were not individually recorded, adjusted or stratified analysis was not feasible. Thyroid malignancy, a pathology within this cohort, is a subject of active molecular research concerning tumor heterogeneity and progression, particularly regarding lipid metabolism and alternative splicing in papillary thyroid carcinoma [31, 32]. However, this oncological literature does not pertain to the anesthetic and neuromonitoring questions addressed here.

Two additional points concern the monitoring itself. Post-resection stimulation for R2/V2 responses was not performed, so the study provides no information on amplitude changes across the dissection, a parameter strongly linked to nerve outcome and the basis for adverse EMG changes. Neuromuscular recovery was also not confirmed with train-of-four monitoring before stimulation, and the interval between rocuronium administration and stimulation for amplitude measurement was not separately logged. While rocuronium induction dose and timing were identical across groups, and neither adjunct acts at the neuromuscular junction, earlier vagus and RLN identification in Group D likely led to earlier stimulation in that group. This proximity to rocuronium administration could have resulted in comparatively more residual block at the time of measurement, potentially contributing to the observed amplitude difference alongside any direct pharmacological effect of dexmedetomidine. Dedicated train-of-four monitoring, synchronized with each stimulation, is necessary to differentiate between these explanations. Propofol and remifentanil were titrated within predefined ranges based on BIS values and clinical need in both groups. However, the actual total or mean intraoperative dose administered to each patient was not recorded—only the infusion rate range was documented, and cumulative dose was not calculated. If titration requirements differed systematically between groups, this could have contributed to the observed differences in recovery time, hemodynamics, and operative conditions, a contribution we cannot quantify here. Finally, our midazolam regimen may not align with practices elsewhere, no dose-response relationship was explored for either drug, and observation was limited to the first postoperative day, thus not capturing later recovery.

Adequately powered, randomized, and blinded multicenter trials, ideally incorporating objective measures of operating conditions, are essential to confirm the reality of the reported differences and their clinical significance for patients.

## Conclusion

In this small, non-randomized, unblinded study of thyroidectomy patients undergoing IONM, the addition of dexmedetomidine to propofol-based TIVA was associated with shorter nerve identification times and smoother early recovery compared to midazolam. EMG amplitudes at both stimulation sites were lower with dexmedetomidine, and bradycardia occurred in 16.7% of patients receiving the drug, though transiently and without consequence in this low-risk cohort. Both regimens yielded interpretable neuromonitoring signals throughout the procedure, with all recorded amplitudes well above the loss-of-signal threshold. Therefore, dexmedetomidine may be a suitable adjunct for IONM-guided thyroid surgery in selected patients, provided cardiac rhythm is closely monitored due to the observed 16.7% incidence of bradycardia. However, the modest, unblinded, non-randomized differences supporting this suggestion require confirmation before influencing clinical practice.

## Supplemental data

Graphical abstract